# Non-response After Gastric Bypass and Sleeve Gastrectomy—the Theoretical Need for Revisional Bariatric Surgery: Results from the Scandinavian Obesity Surgery Registry

**DOI:** 10.1007/s11695-023-06783-0

**Published:** 2023-08-16

**Authors:** Stephan Axer, Eva Szabo, Ingmar Näslund

**Affiliations:** 1 Faculty of Health and Medicine, Örebro University, Campus USÖ, 701 82 Örebro, Sweden; 2 Department of Surgery, Torsby Hospital, Box 502, 685 29 Torsby, Sweden; 3 Department of Surgery, Faculty of Health and Medicine, Örebro University, Campus USÖ, 701 82 Örebro, Sweden

**Keywords:** Revisional bariatric surgery, Sleeve gastrectomy, Gastric bypass

## Abstract

**Background:**

Revisional surgery is a second-line treatment option after sleeve gastrectomy (SG) and gastric bypass (GBP) in patients with primary or secondary non-response. The aim was to analyze the theoretical need for revisional surgery after SG and GBP when applying four indication benchmarks.

**Method:**

Based on data from the Scandinavian Obesity Surgery Registry, SG and GBP were compared regarding four endpoints: 1. excess weight loss (%EWL) < 50%, 2. weight regain of more than 10 kg after nadir, 3. fulfillment of previous IFSO-guidelines, or 4. ADA criteria for bariatric metabolic surgery 2 years after primary surgery.

**Results:**

A total of 60,426 individuals were included in the study (SG: *n* = 7856 and GBP: *n* = 52,570). Compared to patients in the GBP group, more SG patients failed to achieve a %EWL > 50% (23.0% versus 8.5%, *p* < .001), regained more than 10 kg after nadir (4.3% versus 2.5%, *p* < .001), and more often fulfilled the IFSO criteria (8.0% versus 4.5%, *p* < .001) or the ADA criteria (3.3% versus 1.8%, p < 001) at the 2-year follow-up.

**Conclusion:**

SG is associated with a higher risk for weight non-response compared to GBP. To offer revisional bariatric surgery to all non-responders exceeds the bounds of feasibility and operability. Hence, individual prioritization and intensified evaluation of alternative second-line treatments are necessary.

**Graphical Abstract:**

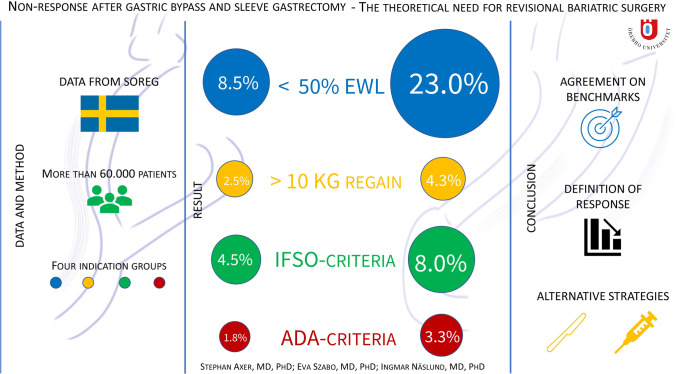

## Introduction

With the increasing number of bariatric procedures performed worldwide, outcomes and indications for revisional procedures have gained more interest in the literature.

As a rule, long-term complications of bariatric operations can be resolved or at least improved by surgical procedures classified as corrective, conversional, or reversal [1]. Discussions revolve around aspects such as timing, patient optimization, type of procedure, and centralization to high volume centers [2].

On the other hand, the surgical treatment of relapse or persistence of obesity and its related diseases is a highly controversial topic. Although the bariatric community has put much effort into introducing scientifically robust guidelines, there is still profound disagreement regarding nomenclature, outcome measures, type of second-line treatment, and the patient’s ability to contribute to treatment results. Poor weight loss results and complication rates after revisional bariatric metabolic surgery have probably contributed to a certain degree of reluctance among some bariatric surgeons [3, 4].

This Swedish study estimates the theoretical need for a surgical second-line treatment after previous bariatric metabolic surgery using indications based on different guidelines and definitions. It is assumed that the previous IFSO and American Diabetes Association (ADA) guidelines regarding the indication for bariatric surgery pertain equally to primary and secondary surgery. Moreover, the study utilized two of the most frequently reported indications for revisional bariatric metabolic surgery based on treatment outcomes.

## Material and Methods

### Data Source and Study Groups

In November 2020, data were retrieved from the Scandinavian Obesity Surgery Registry (SOReg), a nationwide registry described previously [5]. All patients operated with primary GBP or SG at 44 hospitals in Sweden between January 2007 and December 2017 were included in the study and were eligible for a 2-year follow-up. The study was approved by the Regional Ethics Committee (Dnr 2020 02268).

### Indication Groups for Revisional Bariatric Surgery as Second-Line Treatment After Previous Bariatric Metabolic Surgery

Four different groups were defined according to the indication for revisional surgery as second-line treatment after previous GBP or SG. Groups I and II were based on frequently reported indications for revisional bariatric surgery, whereas the criteria for bariatric metabolic surgery according to the IFSO and ADA guidelines were applied in Groups III and IV.

Group I consisted of patients who had a percentage excess weight loss (%EWL) of less than 50% 2 years after their primary bariatric metabolic surgery [6, 7]. Group II comprised patients with increase in weight >10 kg after the nadir, also a frequently reported indication in the literature [7–10]. A prerequisite for weight calculations was attendance at both 1- and 2-year follow-ups. Patients who, after 2 years, still fulfilled the criteria for bariatric metabolic surgery according to the previous IFSO guidelines (BMI > 40 or a BMI 35–40 plus at least one associated medical problem) formed indication Group III [11]. According to the ADA guidelines, bariatric metabolic surgery should be considered in patients “who do not achieve durable weight loss and improvement in associated medical problems (including hyperglycemia) with nonsurgical methods.” Group IV comprised patients with BMI > 30 and pharmacologically treated type-2 diabetes (T2D) at the 2-year follow-up, thereby meeting the criteria for bariatric metabolic surgery as recommended in the ADA guidelines [12].

### Associated Medical Problems

T2D was defined as a glycated hemoglobin A1c (HbA1c) ≥6.5%, fasting blood glucose ≥7 mmol/l, or treatment with antidiabetic medication. The definition of hypertension required ongoing medication. Treatment with continuous positive airway pressure (CPAP) indicated obstructive sleep apnea (OSA). Termination of antidiabetic medication in association with achievement of normal HbA1C and fasting glucose levels was defined as diabetes remission. Cessation of antihypertensive medication or CPAP treatment indicated remission of hypertension and OSA, respectively.

### Data Collection

We collected and analyzed the following variables: age, gender, type of bariatric procedure, associated medical problems (T2D, hypertension, OSA), weight, and BMI. Weight and BMI results were measured as % excess weight loss (%EWL), with the calculation of ideal body weight as that equivalent to a BMI of 25 kg/m2 with excess BMI > 25 kg/m2 ((pre-op BMI − current BMI / pre-op BMI − 25) × 100) as well as % total weight loss (%TWL): ((pre-op weight − current weight / pre-op weight) × 100).

### Statistical Analysis

Data were analyzed using SPSS version 26 (IBM, Armonk, NY). Continuous variables were analyzed using the unpaired Student’s 2-sample *t* test. The *χ*2 test was applied in the statistical analysis of categorical variables. The evaluation of predictors involved both univariate and multivariable logistic regression analysis. In the multivariable analysis, independent variables with more than ten events and *p* values < 0.1 were considered. The selection process for the multivariable analysis followed backward selection. The quality of the prediction model was assessed using the −2log likelihood measure. A significance level of *p* < 0.05 was set for both the baseline variables and the multivariable regression analysis.

## Results

### Baseline

A total of 60,426 patients were included in the study. Most patients had undergone GBP (*n* = 52,570), whereas SG was performed in 7856 individuals. None of the participants required revisional surgery during the study period. Table 1 (part A) displays demographics and patient-specific factors at baseline.

**Table 1:** Pre-operative demographics, associated medical problems, and follow-up data

	Gastric bypass	Sleeve gastrectomy
Number	52,570 (86.7%)	7856 (13.3%)
A: Pre-operative data		
Demographics		
Age (years)	40.9 ± 11.2	41.3 ± 10.8
Female (%)	75.7	80.4
BMI (kg/m^2^)	42.6 ± 5.4	39.6 ± 5.9
Associated medical problems		
OSA	5241 (10.0%)	534 (9.2%)
Hypertension	13,377 (25.5%)	1591 (20.3%)
T2D	10,131 (35.0%)	1054 (21.1%)
B: 2-year follow-up		
Rates and datasets		
Rate	34,133/52,570 (64.9%)	5025/7856 (64.0%)
Complete data on weight/BMI	34,098/34,133 (99.9%)	4992/5025 (99.3%)
Complete data on AMP*	34,103/34,133 (99.9%)	5016/5025 (99.8%)
Complete data on T2D*	17,553/34,133 (51.4%)	2593/5025 (51.6%)
Weight results		
BMI (kg/m^2^)	28.5 ± 4.7	29.7 ± 5.3
%EWL	83.7 ± 35.9	73.2 ± 30.5
%TWL	32.3 ± 8.8	25.6 ± 9.8
Associated medical problems		
OSA	938 (2.7%)	131 (2.6%)
Hypertension	5942 (17.3%)	831 (16.6%)
T2D	2365 (13.0%)	273 (10.2%)

*BMI* body mass index (kg/m^2^), *OSA* obstructive sleep apnea, *T2D* type 2 diabetes, *AMP* associated medical problems, *%EWL* percental excess weight loss, *%TWL* percental total weight loss

*Complete data implies that information regarding treatment and blood values (HbA1C and fasting glucose) were recorded

### 2-Year Follow-up

At the 2-year follow-up, patients in the GBP group had a significantly higher %EWL (83.7 ± 35.9 versus 73.2 ± 30.5, *p* < .001) and %TWL (32.3 ± 8.8 versus 25.6 ± 9.8, *p* < .001). Remission rates of OSA (77.6% versus 73.3%) and T2D (53.5% versus 50.9%) were equal. Hypertension resolved significantly more frequently in the GBP group (41.6% versus 34.8%, *p* < .001). Complete results of the 2-year follow-up are shown in Table 1 (part B).

### Indications for Revisional Bariatric Metabolic Surgery as Second-Line Treatment

In Group I, a significantly higher proportion of patients who underwent SG qualified for surgical second-line treatment due to not achieving an EWL greater than 50% compared to GBP (23.0% versus 8.5%, *p* < .001). The results from both univariate and multivariate analyses are presented in Table 2(part A), with the latter showing that SG is a strong predictor for weight loss below 50% of excess weight (OR 4.46; 95% CI, 4.04–4.93; *p* < .001). Additionally, independent risk factors associated with meeting this criterion include BMI (OR 1.11; 95% CI, 1.10–1.12; *p* < .001), age (OR 1.24; 95% CI, 1.13–1.36; *p* < .001), male gender (OR 1.24; 95% CI, 1.13–1.36; *p* < .001), and T2D (OR 2.00; 95% CI, 1.82–2.19; *p* < .001).

**Table 2:** Characteristics, univariate analyses, and multivariate analyses of Groups I–IV

Characteristic		Univariate analysis		Multivariate analysis	
		Odds ratio (95% CI)	*p* value	Odds ratio (95% CI)	*p* value
A: %EWL of less than 50% (Group I)					
Age (years)	41.4 ± 11.2	-	-	1.24 (1.13–1.36)	< 0.001
BMI	44.4 ± 6.4	-	-	1.11 (1.10–1.12)	< 0.001
Bariatric procedure					
Gastric bypass	2912/34,098 (8.5%)	0.0	REF		
Sleeve gastrectomy	1146/4992 (23.0%)	**3.20 (2.96–3.45)**	**< 0.001**	4.46 (4.04–4.93)	< 0.001
Gender					
Female	2789/30,122 (9.3%)	0.0	REF		
Male	1269/8968 (14.2%)	**1.62 (1.51–1.73)**	**< 0.001**	1.24 (1.13–1.36)	< 0.001
Associated medical problems					
OSA	599/3828 (15.6%)	**1.71 (1.55–1.87)**	**< 0.001**	1.12 (0.99–1.26)	n.s.
Hypertension	1435/10,228 (14.0%)	**1.63 (1.52–1.75)**	**< 0.001**	1.08 (0.98–1.19)	n.s.
T2D	1318/7472 (17.6%)	**2.18 (2.01–2.36)**	**< 0.001**	2.00 (1.82–2.19)	< 0.001
B: Regain of at least 10 kg after nadir (Group II)					
Age (years)	38.1 ± 11.1	-	-	0.97 (0.96–0.98)	< 0.001
BMI	43.6 ± 6.0	-	-	1.05 (1.04–1.07)	< 0.001
Bariatric procedure					
Gastric bypass	795/31,622 (2.5%)	0.0	REF		
Sleeve gastrectomy	205/4721 (4.3%)	**1.81 (1.54–2.11)**	**< 0.001**	2.04 (1.67–2.48)	< 0.001
Gender					
Female	730/28,721 (2.5%)	0.0	REF		
Male	270/8417 (3.2%)	**1.27 (1.10–1.46)**	**0.001**	1.13 (0.91–1.40)	n.s.
Associated medical problems					
OSA	89/3653 (2.4%)	0.89 (0.72–1.11)	0.31	-	-
Hypertension	228/9808 (2.3%)	**0.82 (0.71–0.95)**	**0.009**	1.13 (0.92–1.41)	n.s.
T2D	209/7093 (2.9%)	**1.18 (1.00–1.40)**	**0.054**	1.54 (1.27–1.86)	< 0.001
C: BMI > 40 or a BMI between 35 and 40 in combination with at least one associated medical problem at 2-year follow-up (Group III)					
Age (years)	45.8 ± 11.5	-	-	1.03 (1.02–1.04)	< 0.001
BMI	50.3 ± 6.6	-	-	1.33 (1.31–1.35)	< 0.001
Bariatric procedure					
Gastric bypass	1550/33,337 (4.6%)	0.0	REF	REF	
Sleeve gastrectomy	384/4819 (8.0%)	**1.78 (1.58–2.00)**	**< 0.001**	4.30 (3.60–5.12)	< 0.001
Gender					
Female	1226/29,434 (4.2%)	0.0	REF	REF	
Male	708/8722 (8.1%)	**2.03 (1.85–2.24)**	**< 0.001**	1.08 (0.93–1.24)	n.s.
Associated medical problems					
OSA	470/3761 (12.5%)	**3.21 (2.88–3.59)**	**< 0.001**	1.57 (1.33–1.85)	< 0.001
Hypertension	1016/10,108 (10.1%)	**3.30 (3.01–3.62)**	**< 0.001**	2.29 (1.96–2.69)	< 0.001
T2D	789/7329 (10.8%)	**3.45 (3.09–3.86)**	**< 0.001**	2.58 (2.23–2.98)	< 0.001
D: T2D treated with insulin, oral antidiabetics, or a combination of both, and BMI > 30 (Group IV)					
Age (years)	47.9 ± 10.0	-	-	1.07 (1.05–1.08)	< 0.001
BMI	42.0 ± 5.7	-	-	1.12 (1.10–1.13)	< 0.001
Bariatric procedure					
Gastric bypass	506/5312 (9.5%)	0.0	REF		
Sleeve gastrectomy	133/568 (23.4%)	**2.91 (2.34–3.60)**	**< 0.001**	2.23 (1.78–2.80)	< 0.001
Gender					
Female	344/3789 (9.1%)	0.0	REF	REF	
Male	295/2091 (14.1%)	**1.65 (1.39–1.94)**	**< 0.001**	1.61 (1.34–1.94)	< 0.001
Associated medical problems					
OSA	141/1103 (12.8%)	**1.26 (1.03–1.54)**	**0.023**	0.90 (0.72–1.13)	n.s.
Hypertension	465/3389 (13.7%)	**2.12 (1.76–2.54)**	**< 0.001**	1.42 (1.15–1.75)	0.001
T2D without treatment	181/3490 (5.2%)	0.0	REF	REF	
T2D with other treatment	10/120 (8.3%)	**1.66 (0.86–3.23)**	**0.13**	1.84 (0.93–3.64)	n.s.
T2D treated w/ oral AD	204/1365 (14.9%)	**3.21 (2.60–3.97)**	**< 0.001**	3.16 (2.53–3.95)	< 0.001
T2D treated w/ insulin + oral AD	164/612 (26.8%)	**6.69 (5.30–8.45)**	**< 0.001**	7.00 (5.44–9.00)	< 0.001
T2D treated with insulin	80/293 (27.3%)	**6.87 (5.10–9.24)**	**< 0.001**	8.39 (6.09–11.56)	< 0.001

Bold font indicates variables included in the multivariate regression to identify factors independently contributing to membership in respective category. Table 2 (part D) exclusively comprises patients with T2D at baseline

*BMI* body mass index (kg/m^2^); *OSA* obstructive sleep apnea; *T2D* type 2 diabetes, classified according to SOReg-criteria and ADA-criteria (*SOReg* Scandinavian Obesity Surgery Registry; *ADA* American Diabetes Association); *AD* antidiabetic medication

In Group II, there were significantly more SG patients who experienced weight regain of at least 10 kg after reaching the lowest weight compared to GBP (4.3% versus 2.5%, *p* < .001). The multivariate regression model identified SG as the strongest predictor (OR 2.04; 95% CI, 1.67–2.48; *p* < .001). Similarly, BMI (OR 1.05; 95% CI, 1.04–1.07; *p* < .001), younger age (OR 1.03; 95% CI, 1.02–1.04; *p* < .001), and T2D (OR 1.54; 95% CI, 1.27–1.86; *p* < .001) were identified as independent risk factors for weight regain, as shown in Table 2 (part B).

At the 2-year follow-up, 8.0% of SG patients had a BMI > 40 or a BMI between 35 and 40 plus at least one associated medical problem, compared to 4.5% of the GBP patients (*p* < .001). These patients belonging to Group III thus continued to fulfill the previous IFSO guidelines criteria for bariatric metabolic surgery. In the univariate analysis of potential risk factors for being in Group III and in the multivariate regression model (Table 2, part C), SG emerged as the strongest predictor (OR 4.30; 95% CI, 3.60–5.12; *p* < .001).

Only 2% (equivalent to 639 patients) met the criteria to be included in Group IV. Within this group, 3 patients had de novo T2D. The likelihood of being categorized in this group was doubled for individuals who had undergone SG. However, the most influential risk factor was the presence of T2D at the time of the primary surgery. Additionally, the intensity of the treatment for T2D determined the odds of still meeting the ADA criteria: T2D with oral antidiabetic medication (OR 3.16; 95% CI, 2.53–3.95; *p* < .001), T2D with oral antidiabetic medication plus insulin (OR 7.00; 95% CI, 5.44–9.00; *p* < .001) and T2D with insulin (OR 8.39; 95% CI, 6.09–11.56; *p* < .001) (Table 2, part D).

## Discussion

Three problems that have long plagued the bariatric surgery community: disagreement on outcomes, how weight loss should be calculated, and definition of the effect on associated medical problems. These have prevented meaningful evaluation and comparison of treatment results [13, 14]. In 2015, Brethauer et al. published “Standardized Outcomes Reporting in Metabolic and Bariatric Surgery” as an attempt “to provide guidance to authors and editors who write, review, and publish manuscripts focusing on bariatric and metabolic surgery.” This article contributed to improvements in the quality and comparability of reports on primary bariatric metabolic surgery. However, three aspects remained unanswered: 1. interpretation of results, 2. benchmarks for escalation of therapy, and 3. standardization of outcomes of second-line treatment. Weight loss has traditionally been labeled as “success,” “failure,” or “regain.” This terminology was questioned, and a more descriptive nomenclature proposed, *i.e*., primary responder (“success”), primary non-responder (“failure,” “weight loss failure,” or “insufficient weight loss”), and secondary non-responder (“weight regain”) that in turn has also been questioned [7, 15]. According to the results of a survey of 460 surgeons by Mahawar et al., most bariatric surgeons defined an EWL > 50% as a “successful response.” Specific criteria regarding the resolution of metabolic-associated medical problems were used by less than 50% of respondents. Definitions of “primary non-responders” or “secondary non-responders” were inconsistent [16]. These results are in line with several other publications [8, 9, 17]. Given the lack of consensus within the bariatric community regarding the evaluation and ranking of treatment outcomes, the decision to expand treatment through revisional surgery remains challenging. Consequently, the decision to escalate therapy is left to the discretion of the individual surgeon.

The aim of this study was to scrutinize the need for revisional bariatric metabolic surgery when applying four different indication categories: Group I, an EWL of less than 50%; Group II, regain of 10 kg or more in weight after reaching the nadir; Group III, application of IFSO guidelines’ criteria; and Group IV, application of ADA guidelines’ criteria. The indications in Groups I and II are frequently applied in the literature [7–10], and 12.1% of patients included in our study could be placed in one or other of these two indication groups. After adjustment for confounding factors, it was 4 times more likely for SG patients to fulfill these criteria for revisional bariatric surgery after 2 years compared to GBP patients.

It is unusual to utilize the IFSO or ADA guidelines’ criteria for revisional bariatric metabolic surgery. However, neither the IFSO nor the ADA recommendations is explicitly meant for primary bariatric metabolic surgery. Continuing to meet IFSO guidelines’ criteria has previously been applied by several authors [18–20]. Two years after bariatric metabolic surgery, 8.0% of SG patients had a BMI > 40 or a BMI 35–40 plus at least one associated medical problem (Group III) compared to 4.5% among the GBP patients. Prior to primary surgery, approximately 58% of the SG patients and about 84% of the GBP patients met the previous IFSO criteria. To our knowledge, the ADA guidelines’ criteria have not been applied as an indication for revisional bariatric metabolic surgery. A total of 2.0% of patients with a BMI > 30 and pharmacologically treated T2D 2 years after SG or GBP formed Group IV in this study. SG patients had a twofold higher probability of being in Group IV compared to GBP. In the present study, the rates of T2D remission were in line with the results published in a systematic review and meta-analysis of randomized trials comparing SG and GBP [21]. One predictive factor for continuing to meet the ADA criteria for bariatric metabolic surgery after primary surgery is diabetes treatment that depends on insulin. It is well known that low intensity diabetes treatment, short duration of T2D, and satisfactory glucose control are decisive factors for the probability of diabetes resolution after bariatric metabolic surgery [22–24].

Regardless of the criteria used to identify patients requiring revisional bariatric surgery, SG was clearly a risk factor for weight non-response. This can be interpreted as indirect confirmation of the results of two randomized trials [25, 26]. We had no intention to question SG as a technique per se nor was our intention to develop a risk model for the need for revisional surgery, identify contributing risk factors for non-response, or present improvement suggestions regarding patient selection and surgical techniques. The idea was simply to pose the question: how significant is the theoretical need for revisional bariatric metabolic surgery after previous SG or GBP if we adhere to certain criteria?

Considering the resources available in Sweden, it is not practical to offer revisional bariatric metabolic surgery to all patients identified within the four indication groups mentioned in this study. The management of obesity should consistently recognize its chronic nature, necessitating long-term care. Ensuring universal access to treatment, regardless of the chosen approach, is of importance. To tackle this challenge, a roadmap should encompass key action points: establishing agreement on treatment benchmarks, developing evaluation tools for result interpretation, and assessing alternative non-surgical treatment strategies. The responsibility lies with the bariatric community to establish a consensus on indications for revisional bariatric surgery, while simultaneously allowing for individual assessments and deviations from the established guidelines. It is frequently observed that patients who potentially meet the criteria for revisional bariatric metabolic surgery 2 years after their primary procedure report satisfaction with the favorable outcomes, including the resolution of OSA, successful childbirth, improved quality of life, and alleviation of back pain. The intended outcomes of primary bariatric metabolic surgery must be well-defined and crystal clear, otherwise critical evaluation of individual results is impossible. Multidisciplinary assessment of all patients before primary and secondary interventions is highly desirable [27, 28]. Proactive interdisciplinary follow-up is fundamental to identify primary and secondary non-responders [29]. Courcoulas et al. demonstrated an association between the “magnitude and direction/slope of the initial loss” and long-term weight trajectories, stressing the importance of “enhanced and early (Years 1-2) postoperative efforts to optimize short-term weight loss trajectory directions” [30]. The sooner secondary treatment is initiated, the better the results in the long run. Similarly, the results of a study by Brissman et al. suggest the importance of early reintervention, showing that percentage weight loss during the first postoperative year was predictive of “surgical treatment failure 5 years after surgery” [31]. Adjuvant pharmacological therapy in the early period after bariatric metabolic surgery may enhance treatment results [32–34]. Compared to primary surgery, the risk for perioperative complications is higher in revisional surgery [4]. Non-surgical treatment options should be thoroughly evaluated and should be considered when deciding on reintervention or therapy escalation [29]. The major challenge is to establish a robust scientific foundation for the solution of this complex problem. It is well established that patients with advanced obesity and associated medical problems do not benefit as much from changes in lifestyle or intensive medical therapies compared to the benefits of primary bariatric metabolic surgery. It appears that this is not directly applicable to secondary treatment approaches. The superiority of revisional bariatric surgery over non-surgical interventions has not been confirmed. Randomized trials studying second-line treatment of obesity and related diseases are lacking despite the number of patients concerned.

## Limitation

The study had a retrospective design and used registry data, which comes with certain limitations. Furthermore, there is a lack of agreement among bariatric surgeons regarding the criteria for revisional bariatric surgery or other second-line treatments. Nevertheless, we performed this study specifically to address this issue and do not consider it a limitation. We acknowledge that applying the new IFSO guidelines, which were published in autumn 2022, to our study would have led to even more patients being eligible for second-line treatment.

## Conclusion

This study focused on the need for revisional bariatric metabolic surgery using four indication categories. SG patients were more likely to require revisional surgery compared to GBP patients. Limited resources make it impractical to offer revisional surgery to all eligible patients. Key actions include establishing treatment benchmarks and evaluating non-surgical treatment options.
